# Effect of single-injection thoracic paravertebral block with liposomal bupivacaine on the quality of recovery after thoracic surgery: a protocol for an open-label, randomized controlled trial

**DOI:** 10.3389/fmed.2025.1734331

**Published:** 2026-01-12

**Authors:** Wenru Ma, Li Wang, Haiping Zhang, Meijia Zou, Shiran Feng, Yinhuan Liu, Peng Wang

**Affiliations:** 1 Department of Anesthesiology, The Affiliated Hospital of Qingdao University, Qingdao, China; 2 Department of Clinical Medicine, Qingdao University, Qingdao, Shandong, China

**Keywords:** analgesia, liposomal bupivacaine, nerve block, pain, paravertebral block, quality of recovery, thoracic surgical procedures

## Abstract

**Background:**

Postoperative pain management plays a crucial role in determining the quality of recovery following thoracic surgery. However, achieving high-quality postoperative analgesia that facilitates satisfactory recovery remains challenging due to suboptimal pain control and side effects associated with conventional analgesic strategies. This study aims to evaluate the effect of single-injection thoracic paravertebral block with liposomal bupivacaine on the overall quality of recovery in patients undergoing thoracic surgery.

**Methods:**

This single-center, open-label, randomized controlled trial will enroll 162 patients scheduled for video-assisted thoracic surgery. Participants will be randomly allocated in a 1:1:1 ratio to three groups (*n* = 54 each): Group L will receive ultrasound-guided thoracic paravertebral block with liposomal bupivacaine; Group R will receive ropivacaine-based paravertebral block combined with opioid-based patient-controlled intravenous analgesia; and Group P will receive opioid-based patient-controlled intravenous analgesia alone. The primary outcome is the quality of recovery assessed by patient-reported Quality of Recovery-15 scores on postoperative days 1–3. Between-group differences will be analyzed using generalized estimating equations. Secondary outcomes include: pain-free interval; resting and cough-induced pain intensity at 6, 12, 24, 48, and 72 h; worst pain scores at 24, 48, and 72 h; perioperative opioid use; time to first rescue analgesia and times of request; patient confidence in coughing and ambulation; anxiety and sleep quality at 24, 48, and 72 h; patient satisfaction; postoperative nausea and vomiting incidence/severity and rescue antiemetic use. Safety outcomes comprise block-related complications and postoperative complications. All adverse events will be documented throughout the study.

**Discussion:**

This study aims to provide high-quality evidence on the efficacy of a single-injection thoracic paravertebral block with liposomal bupivacaine for post-thoracic surgery analgesia, compared with opioid-based intravenous analgesia alone and the combination of ropivacaine paravertebral block with systemic opioid analgesia.

**Clinical trial registration:**

Identifier: ChiCTR2500107787.

## 1 Introduction

Although contemporary thoracic surgery is performed with video-assisted thoracoscopic surgery (VATS), which offers a minimally invasive approach, patients who undergo such procedures still experience moderate to severe postoperative pain, that can be attributed to rib retraction, intercostal nerve injury, and the irritation of the pleura by drainage tubes (1–4). This discomfort may negatively impact both postoperative recovery and quality of life (5). Efficient cough and expectoration is integral to the mitigation of pulmonary infection rates, and the commencement of early and vigorous ambulation is instrumental in reducing the incidence of lower extremity deep vein thrombosis (1, 6, 7), however, these necessary postoperative recoveries are often accompanied by an exacerbation of pain. Therefore, optimal postoperative pain management is crucial for enhancing the quality of patient recovery.

Postoperative pain management employs various approaches—including epidural, intravenous, regional, and local analgesia—with differing efficacy levels. In Canada, epidural analgesia remains preferred for open thoracic surgery, while 41% of clinicians use it for thoracoscopic procedures and 27% favor intravenous analgesia (8). Catheters and opioids, despite limitations, are indispensable in Enhanced Recovery After Surgery (ERAS)-aligned pain management (9–11). Catheter-based techniques carry risks of occlusion, dislodgement, and infection. Although established in practice, epidural analgesia may cause unavoidable discomfort, neurological injury, hematoma, hypotension, urinary retention, and cardiovascular complications (8, 12–14). Intravenous analgesia usually relies on opioids, which frequently cause postoperative nausea and vomiting (15, 16). Therefore, an ideal postoperative pain management strategy should eliminate both catheters and opioids.

Prospect guidelines for video-assisted thoracoscopic surgery explicitly advocate for the use of thoracic paravertebral nerve block (PVB) as the preferred modality for postoperative pain management following thoracoscopic surgery (7). Studies have shown that thoracic PVB is comparable to epidural analgesia in terms of pain relief efficacy (1, 17–19). However, effective postoperative analgesia requires both sufficient intensity and duration. Single-injection nerve blocks are limited by the short duration of conventional local anesthetics. Liposomal bupivacaine represents a novel extended-release local anesthetic formulation with an established safety profile. Utilizing multivesicular liposome technology, the agent encapsulates bupivacaine within phospholipid vesicles (20). This structural design facilitates progressive drug release, maintaining stable plasma concentrations and thereby enabling prolonged, high-quality analgesia. Its distinct pharmacokinetic profile supports its potential in postoperative pain management, making single-injection regional anesthesia a feasible standalone analgesic strategy.

This study will evaluate a novel analgesic strategy combining paravertebral block with liposomal bupivacaine in thoracic surgery. The approach is expected to provide analgesic efficacy comparable to that of continuous catheter-based techniques while substantially reducing complication rates, thereby enhancing postoperative recovery quality. These findings will provide crucial evidence supporting the implementation of catheter-free and opioid-free pain management protocols in postoperative care.

## 2 Methods

This protocol adheres to the Standard Protocol Items: Recommendations for Interventional Trials (SPIRIT) guidelines.

### 2.1 Study design and patients

**Figure 1:**
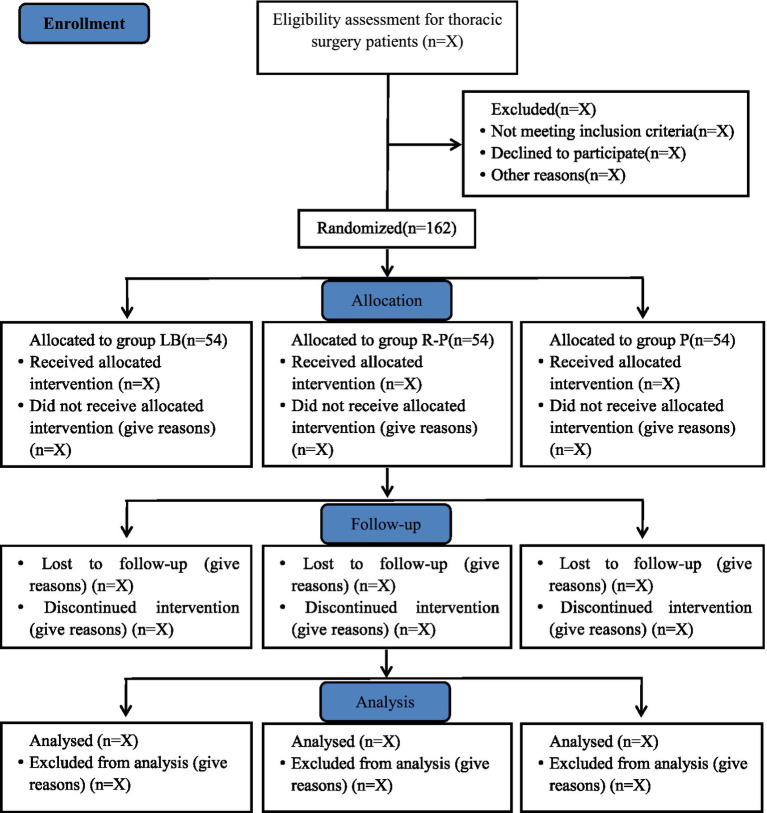
Study flow diagram.

This study is a single-center, prospective, randomized, open-label, parallel-group clinical trial. The trial will be conducted at the Affiliated Hospital of Qingdao University, enrolling a total of 162 patients. Recruitment is scheduled to take place from 18th August 2025 to 7th May 2026. The study flow diagram is shown in Figure 1.

### 2.2 Inclusion criteria

Eligible participants will be adults aged 18 to 90 years scheduled for unilateral thoracoscopic surgery, with an American Society of Anesthesiologists (ASA) physical status of I to III, who provide written informed consent.

### 2.3 Exclusion criteria

Exclusion criteria will include:

- Known allergy to local anesthetics, nonsteroidal anti-inflammatory drugs, or opioids;
- Preexisting chronic pain of thoracic or back origin;
- Contraindications to regional puncture such as coagulopathy, local infection or inflammation;
- Psychiatric disorders;
- History of ipsilateral thoracotomy;
- Referral for thoracotomy;
- History of chronic opioid use or substance abuse;
- Bilateral surgery.

### 2.4 Primary outcome

The primary outcome of this study is the composite score of the Quality of Recovery-15 (QoR-15) score at 24, 48, 72 h postoperatively. The QoR-15 is a multidimensional patient-reported instrument designed to evaluate functional recovery after surgery. It comprises five domains: physical comfort (5 items), physical independence (2 items), pain (2 items), emotional state (4 items), and psychological support (2 items). Each item is rated on a 10-point scale, ranging from 0 (never) to 10 (all of the time) (scores for negative items are reversed). Higher QoR-15 scores indicate better quality of recovery after surgery.

### 2.5 Secondary outcomes

Secondary outcomes will include the pain-free interval (defined as the time from the end of surgery to the first onset of pain), resting and cough-evoked pain Visual Analog Scale (VAS) scores (ranging from 0, no pain, to 10, worst imaginable pain) assessed at 6, 12, 24, 48, and 72 h postoperatively; maximum pain scores at 24, 48, and 72 h; intraoperative and total postoperative opioid consumption; time from the end of surgery to first request for rescue analgesia and the number of such requests; anxiety and sleep quality (using numeric rating scores, NRS, 0–10, higher indicating greater anxiety/ greater sleep) at 24, 48, and 72 h; patient satisfaction with analgesia measured using an NRS (0–10, higher indicating greater satisfaction); incidence and severity of postoperative nausea and vomiting (PONV), graded on a 4-point scale (0 = none, 1 = nausea only, 2 = 1–3 vomiting episodes, 3 = ≥4 episodes) and the number of rescue antiemetic administrations at 24, 48, and 72 h; as well as patient confidence in coughing and ambulation, both assessed using an NRS (0 = no confidence to 10 = full confidence). All postoperative assessments will be performed daily at 17:00 until 3 days after surgery. The following outcomes will be recorded prior to patient discharge: the incidence of pneumothorax or hemothorax during the entire postoperative period, total postoperative drainage volume, and length of hospital stay.

### 2.6 Safety outcomes

Safety outcomes will include the adverse events related to regional nerve blockade, such as allergic reactions, total spinal anesthesia, severe hypotension, malignant arrhythmias, and cardiac arrest, as well as postoperative complications including urinary retention, puncture site infection, pulmonary infection, deep vein thrombosis, pulmonary embolism, and atelectasis. All adverse events will be recorded and assessed throughout the study period.

### 2.7 Randomization and blinding

This open-label, three-arm randomized trial will assign 162 participants in a 1:1:1 ratio (Group L, Group R, and Group P, with 54 participants per group). An open-label design will be adopted due to fundamental differences in postoperative pain management protocols among the three groups. To facilitate protocol-adherent adjustments in analgesia, both investigators and participants will not be blinded to treatment allocation. Nevertheless, postoperative outcome assessments will be performed by trained nurses who are not involved in the intervention procedures to minimize potential bias.

### 2.8 Anesthetic and study interventions

All patients will fast for at least 8 h and refrain from clear fluids for at least 4 h prior to surgery. In the operating room, following the establishment of peripheral intravenous access, patients will receive oxygen via face mask at 5 L/min and undergo continuous monitoring of vital signs, including non-invasive blood pressure, percutaneous arterial oxygen saturation (SpO₂), heart rate, respiratory rate, and bispectral index (BIS). Under aseptic conditions, a radial arterial catheter will be inserted for continuous invasive arterial pressure monitoring. Anesthesia induction will be performed using propofol (2 mg/kg), sufentanil (0.5 μg/kg), and rocuronium (0.6 mg/kg). After induction of anesthesia, adequate topical anesthesia of the larynx and upper trachea will be achieved using lidocaine. Following preoxygenation, a double-lumen endobronchial tube will be placed. Correct positioning and depth of the double-lumen tube will be confirmed before initiating mechanical ventilation. The patient will then be repositioned into the lateral decubitus position with the surgical side uppermost.

A single senior anesthesiologist will perform ultrasound-guided thoracic paravertebral blocks under sterile conditions for all patients in groups L and R. The target injection levels are T4 and T6. For group L, under real-time ultrasound guidance and after confirming correct needle tip position, 10 mL of liposomal bupivacaine (133 mg/20 mL) (21) will be injected at each of the T4 and T6 levels according to a standardized technique, for a total volume of 20 mL. For group R, under identical ultrasound-guided and aseptic conditions, 10 mL of ropivacaine (0.5%) will be injected at each of the T4 and T6 levels, for a total of 20 mL. Patients in group P will not receive any nerve block. Following surgery, patient-controlled intravenous analgesia (PCIA) will be prepared for patients in groups R and P, but not for those in group L. Each PCIA pump will be prepared with a total volume of 100 mL, containing sufentanil and 8 mg of ondansetron. The total sufentanil dose will be calculated as 2 μg per kilogram of body weight. The background infusion will be set at a rate of 0.04 μg/kg/h. Additionally, the PCIA protocol will be configured with a bolus dose of 0.04 μg/kg and a lockout interval of 15 min to ensure medication safety.

An emergency response team will be established in this study to promptly manage patients with breakthrough severe pain and nausea/vomiting, ensuring rapid symptom relief. The acute pain management protocol will include acetaminophen and oxycodone: initially, 1 g of intravenous acetaminophen will be administered every 6 h (maximum daily dose not exceeding 4 g). If the patient experiences intolerable pain (Visual Analog Scale score > 4) for more than 30 min, or if the maximum daily dose of acetaminophen has been reached, oral oxycodone 5 mg will be administered instead, with dose adjustments within the safe range as needed. Subsequent doses may be repeated every 4 to 6 h under close monitoring and strict control of dosage variations. For participants in groups R and P using PCIA, rescue analgesia will be implemented according to a tiered protocol. If intolerable pain occurs despite the preset continuous sufentanil background infusion, PCIA bolus doses will be administered as the first-line rescue strategy. Should pain remain uncontrolled after reaching the maximum allowable bolus dose, the subsequent, predefined rescue analgesic protocol will then be initiated. For acute episodes of severe nausea and vomiting, potential underlying causes will first be assessed to exclude gastrointestinal symptoms resulting from other primary conditions. Once other causes are ruled out, ondansetron 4 mg will be administered intravenously as rescue therapy (maximum daily dose not exceeding 16 mg). Dosage adjustments may be made based on symptom severity within established safety limits.

### 2.9 Data collection and monitoring

Data collection will encompass a range of patient characteristics, including age, sex, height, weight, body mass index (BMI), ASA physical status, history of alcohol use and smoking, and Zubrod performance status (a preoperative measure of functional capacity and tolerance to treatment). Additional variables include percent predicted forced expiratory volume in 1 s (FEV1%), history of chronic obstructive pulmonary disease, cardiac conditions (including hypertension, coronary artery disease, previous myocardial infarction, and arrhythmias), diabetes, hepatic insufficiency, and renal insufficiency. Surgical details will encompass procedure type (1-wedge resection, 2-segmentectomy, 3-lobectomy), number of port sites (1-uniportal, 2-biportal), laterality (1-left, 2-right), duration of surgery, intraoperative blood loss, and duration of chest tube retention. All pertinent data will be meticulously recorded in case report forms (CRFs) and subsequently inputted into an electronic database under the vigilant supervision of the principal investigator (Table 1). An independent Data Monitoring Committee (DMC) will be tasked with the ongoing oversight of the data collection process. Upon completion of data registration, the electronic database will be safeguarded. De-identified datasets will then be forwarded to an independent statistician for comprehensive analysis in accordance with a predefined statistical plan. Any serious adverse events (SAEs), whether or not they are related to the study medication (e.g., persistent hemodynamic instability), must be promptly reported to the principal investigator. In such instances, the perioperative care team is obligated to implement necessary measures to safeguard participant well-being. Furthermore, these SAEs must be communicated to the DMC within a 24-h window for thorough discussion and to determine if any adjustments to the study interventions or the study’s discontinuation are warranted.

**Table 1:** Schedule of patients enrolment, study interventions, and outcome assessment.

Time point	Study period								
	Enrollment	Allocation	Post-allocation						Close-out
	Pre-op visit	Pre-op 1 day	Intra-op	6 h post-op	12 h post-op	24 h post-op	48 h post-op	72 h post-op	Discharged
Patient enrolment									
Eligibility criteria	×								
Written informed	×								
Consent									
Demographic data	×								
Baseline characteristics	×								
Randomization/allocation		×							
Study interventions									
Liposomal bupivacaine			×						
Ropivacaine & PCIA			×						
PCIA			×						
Outcome assessment									
QoR-15						×	×	×	
Pain-free interval				×	×	×	×	×	
VAS for pain at rest				×	×	×	×	×	
VAS for pain during cough				×	×	×	×	×	
Maximum VAS for pain						×	×	×	
First rescue analgesia				×	×	×	×	×	
Times of rescue analgesia				×	×	×	×	×	
Opioid consumption			×						×
NRS for anxiety						×	×	×	
NRS for sleep quality						×	×	×	
Analgesic Satisfaction									×
Incidence of PONV						×	×	×	
Severity of PONV						×	×	×	
Times of rescue antiemetic						×	×	×	
Confidence in coughing						×	×	×	
Confidence in ambulation						×	×	×	
Pneumothorax									×
Hemothorax									×
Drainage volume									×
Length of hospital stay									×
Adverse events									
Allergic			×	×	×	×	×	×	×
Total spinal anesthesia			×	×	×	×	×	×	×
Hypotension			×	×	×	×	×	×	×
Malignant arrhythmias			×	×	×	×	×	×	×
Cardiac arrest			×	×	×	×	×	×	×
Urinary retention			×	×	×	×	×	×	×
Puncture site infection			×	×	×	×	×	×	×
Pulmonary infection			×	×	×	×	×	×	××
Deep vein thrombosis			×	×	×	×	×	×	×
Pulmonary embolism			×	×	×	×	×	×	×
Atelectasis			×	×	×	×	×	×	×

According to SPIRIT, statement of defining standard protocol items for clinical trials.

Pre-op, preoperative; Intra-op, intraoperative; Post-op, postoperative; QoR-15, quality of recovery-15 scale; VAS, visual analogue scale; NRS, numerical rating scale; PCIA, patient-controlled intravenous analgesia pump; PONV, postoperative nausea and vomiting.

### 2.10 Sample size calculation

The primary outcome will be the QoR-15 score, evaluated at 24, 48, and 72 h after surgery. Based on previously published data, the minimal clinically important difference (MCID) for the QoR-15 is set at 8 points (22), with an estimated standard deviation (SD) of 12 (23). Using an independent-sample *t*-test calculation with a two-sided alpha of 0.05 and 80% power (beta = 0.2), an initial sample size of 36 patients per group will be estimated to detect a statistically significant difference in mean QoR-15 scores. However, because the trial involves three groups and planned pairwise comparisons, a Bonferroni correction will be applied, adjusting the significance threshold to 0.017 (0.05/3). Additionally, since the primary outcome entails repeated measures over three time points, the sample size will be adjusted using an intraclass correlation coefficient (ICC) of 0.36, derived from pilot data. After these adjustments, a minimum of 48 participants per group will be required. The final sample size was calculated using the following formula for repeated measures: The formula is $n=\frac{{\left(Z_{1-a/2}+Z_{1-\beta }\right)}^{2}\times (1+(\kappa -1)\rho )\times \sigma^{2}}{\Delta^{2}\times (1-\rho )}$ *n* is the sample size required per group, κ is the number of repeated measurements (3 in this study), σ is the standard deviation (12), Δ is the minimal clinically important difference (8), and σ is the intraclass correlation coefficient (0.36). This number will then be inflated by 10% to account for potential attrition, loss to follow-up, or major missing data, resulting in a final sample size of 54 patients per group. Thus, a total of 162 patients (54 per group) will be enrolled.

### 2.11 Statistical analysis

The normality of continuous variables will be assessed using the Shapiro–Wilk test. Data following a normal distribution will be reported as mean (standard deviation, SD), while non-normally distributed data will be presented as median (interquartile range, IQR). Binomial variables will be expressed as proportions. The correlation structure will be selected based on the quasi-likelihood under the independence model criterion (QIC). Data distribution will determine the use of either a normal or gamma distribution with identity or log-link functions. The primary hypothesis of this study is that group L can demonstrate a superior quality of postoperative recovery compared to both control groups, with the difference being both statistically and clinically significant. A generalized estimating equation (GEE) model will be used to analyze repeated measures of continuous variables accounting for within-subject correlations. Continuous variables measured at a single time point will be analyzed using *t*-tests or Mann–Whitney U tests depending on normality and categorical data will be analyzed using the χ^2^ test. Post-hoc pairwise comparisons are strictly contingent on a significant overall between-group difference and *p*-values are adjusted using the Bonferroni method and set at 0.017. For longitudinal outcomes, this correction governs testing at both the individual time-point and overall treatment effect levels, ensuring robust control of the type I error rate. Statistical significance will be defined as *p* < 0.05.

All study outcomes will be analyzed in the modified intentiontotreat (mITT) population, including all patients who are randomized, received the assigned intervention, and completed all or part of the postoperative follow-up assessments. Patients will be included in the analysis according to their original allocation. To evaluate the robustness of the primary outcome and its sensitivity to protocol deviations, a per-protocol (PP) analysis will be performed as a sensitivity analysis to verify the reliability of the findings from the intention-to-treat analysis. Multiple testing correction will be applied to primary and secondary outcome measures where applicable. As a theoretical principle, this adjustment is generally not applied to safety indicators. Odds ratios (OR) and corresponding 95% confidence intervals (CI) will be reported where appropriate. No interim analysis will be planned. Missing data will not be imputed. Statistical analyses will be performed using SPSS (version 25.0; IBM SPSS). A two-sided *p*-value < 0.05 will indicate statistical significance, except where false discovery rate (FDR) corrections apply.

### 2.12 Patient and public involvement

Patients and the public will not participate in the study’s design, recruitment, conduct, or reporting. Study results will be shared with participants via email.

## 3 Discussion

This prospective, open-label, randomized controlled trial plans to enroll 162 adult patients at our center. It is designed to evaluate the effect of a single ultrasound-guided paravertebral block using liposomal bupivacaine on postoperative recovery quality compared to conventional opioid-dependent intravenous analgesia and an established high-quality multimodal analgesia protocol. The trial will be conducted in accordance with the Consolidated Standards of Reporting Trials (CONSORT) guidelines. In addition to assessing overall recovery quality, the study will address three key clinical questions: whether the intervention provides sufficient baseline analgesia; whether it independently meets current standards for comprehensive pain management; and whether it confers significant clinical benefits beyond analgesia. The primary outcome is the overall quality of recovery, as measured by QoR-15 score at 24, 48, and 72 h after surgery. Secondary outcomes include multiple pain management quality assessments (such as pain-free interval and pain scores under various conditions), total consumption of sufentanil during surgery and hospitalization, anxiety and sleep quality scores at 24, 48, and 72 h postoperatively, the incidence and severity of PONV along with the use of rescue antiemetics at the same intervals, and patient confidence in coughing and ambulation assessed using an NRS. All postoperative evaluations will be performed daily at 17:00 until 3 days after surgery. Additional recorded outcomes include the incidence of pneumothorax or hemothorax, total postoperative drainage volume, and length of hospital stay.

The distinctive feature of the group L in this trial is its independence from both indwelling catheters and opioid reliance, representing a key divergence from conventional analgesic strategies. This approach may demonstrate advantages by circumventing drawbacks associated with catheter placement and opioid dependence. The primary objective of this design is to directly evaluate the impact of a single-injection thoracic paravertebral block using liposomal bupivacaine on the quality of postoperative recovery, with particular emphasis on the quality of postoperative pain management provided by this strategy. The three-arm trial design enables comparison across two distinct dimensions. The first comparator is the most fundamental opioid-dependent PCIA, representing baseline postoperative pain management. When administered within safe dosing parameters, PCIA can meet essential analgesic requirements for pain relief. The second comparator combines PCIA with a ropivacaine-based thoracic paravertebral block, constituting a more advanced pain management protocol designed to achieve comprehensive analgesia. This experimental design addresses two fundamental questions: whether the single-injection thoracic PVB using liposomal bupivacaine can fulfill the basic requirements of postoperative pain management, and what level of analgesic efficacy it can provide.

Conventional analgesic agents, including intravenous analgesics and local anesthetics, have half-lives insufficient to cover the entire postoperative recovery period. As a result, catheter-based techniques—such as continuous epidural, intravenous, or regional nerve block analgesia—remain widely used in postoperative pain management, despite introducing risks and inconveniences related to catheter placement. Liposomal bupivacaine represents a novel long-acting, sustained-release local anesthetic. The drug is encapsulated within multivesicular phospholipid vesicles, enabling controlled and prolonged release of bupivacaine. Pharmacodynamically, peak plasma concentrations are typically achieved within 30 min after local injection, with analgesic effects lasting up to 72 h (20). Notably, ERAS guidelines emphasize that postoperative analgesia should be initiated preemptively—before pain onset—and maintained effectively (24). Therefore, liposomal bupivacaine emerges as a promising option that may potentially revolutionize postoperative pain management paradigms.

The analgesic efficacy and sustained duration of liposomal bupivacaine have been demonstrated in clinical settings. A study involving local infiltration after hemorrhoidectomy showed that patients receiving liposomal bupivacaine experienced a median time of 14.2 h before requiring supplemental analgesia, compared to only 1.2 h in the control group—reflecting both its potent analgesic effect and significantly prolonged duration of action (25). When used for bilateral transversus abdominis plane (TAP) blocks following cesarean delivery, liposomal bupivacaine significantly enhanced the quality of postpartum recovery and markedly reduced postoperative opioid consumption (26, 27). Furthermore, this strategy provided a complete pain-free period of at least 15 h post-surgery (26). Regarding safety, multiple studies have reported no signs of local anesthetic systemic toxicity (LAST) associated with the local application of liposomal bupivacaine. Importantly, research in obstetric populations confirmed that plasma concentrations of bupivacaine remained within safe limits for both mothers and newborns (28). Therefore, liposomal bupivacaine exhibits a highly favorable safety profile when administered via local infiltration.

PVB is recommended as the preferred analgesic technique for video-assisted thoracoscopic surgery (VATS) in prospective guidelines, owing to its demonstrated advantage (7). PVB has been associated with lower incidences of hypotension and urinary retention compared to epidural analgesia, and the puncture pain associated with PVB is also significantly lower than that of the epidural route (19). Moreover, due to its technical characteristics, PVB can be administered unilaterally according to the specific surgical site. And, compared to PCIA that utilizes opioids, PVB has been shown to markedly reduce the incidence of PONV (29, 30). It also significantly decreases the frequency of postoperative emergency analgesia demands and the opioid consumption (30).

As recommended by clinical guidelines (24, 31, 32), postoperative analgesia should follow an opioid-sparing strategy, which is also a key advantage of multimodal analgesia. This approach not only improves the quality of pain control but also reduces opioid-related adverse effects. The aim of this prospective randomized controlled trial is to combine paravertebral block (PVB) technique with liposomal bupivacaine to investigate the impact of a single-injection ultrasound-guided PVB using liposomal bupivacaine on the quality of postoperative recovery. No placebo control for the purpose of balancing interventions will be included, so as to directly reflect the true clinical effect and value of this strategy. Furthermore, among the secondary outcomes, we plan to specifically analyze the postoperative analgesic quality of this strategy and its contribution to reducing adverse effects such as nausea and vomiting, thereby comprehensively evaluating the clinical significance of this catheter-free and opioid-sparing postoperative pain management approach. By comparing with two reference groups—patients receiving PCIA alone and those receiving PCIA combined with ropivacaine-based PVB—this study will assess the extent of improvement in postoperative recovery quality achievable with ultrasound-guided single-injection PVB using liposomal bupivacaine. Our hypothesis posits that a catheter- and opioid-free pain management strategy—enabled by the sustained-release profile of liposomal bupivacaine and high-precision ultrasound-guided thoracic paravertebral blockade—can provide effective analgesia after thoracic surgery. This RCT will help advance the transformation of pain management paradigms in real-world clinical practice.

## 4 Limitations

This study will have several limitations. First, the trial will be designed to directly reflect the real-world performance of each analgesic strategy and therefore will not incorporate placebo controls. The fundamental differences among the interventions necessitate an open-label design, which may introduce potential biases in subjective outcomes, such as detection bias and reporting bias. Second, this will be a single-center clinical trial with a relatively small sample size, which may limit the generalizability of the findings. Larger, multicenter studies will be needed to validate these results. Third, the implementation of strict inclusion and exclusion criteria will exclude certain patient subgroups, which may further restrict the applicability of our findings to broader populations. Future research will need to focus on evaluating the safety and efficacy of this strategy in more diverse and representative patient cohorts.

## 5 Conclusion

This randomized controlled trial aims to provide rigorous evidence regarding the efficacy and safety of a single-injection thoracic paravertebral block using liposomal bupivacaine for post-thoracotomy analgesia. The study will specifically evaluate the quality of analgesia provided by this catheter- and opioid-free strategy in specific patient populations. Additionally, we will observe its impact on other postoperative recovery indicators and the incidence of adverse effects such as PONV. Our findings are expected to facilitate broader clinical adoption of this approach, thereby enhancing postoperative recovery outcomes. Future research should continue to explore the long-term benefits and broader applicability of this strategy across different surgical populations.

## Data Availability

The raw data supporting the conclusions of this article will be made available by the authors, without undue reservation.
